# CPNE7-Induced Autophagy Restores the Physiological Function of Mature Odontoblasts

**DOI:** 10.3389/fcell.2021.655498

**Published:** 2021-04-26

**Authors:** Yeoung-Hyun Park, Chul Son, You-Mi Seo, Yoon Seon Lee, Alix Har, Joo-Cheol Park

**Affiliations:** ^1^Laboratory for the Study of Regenerative Dental Medicine, Department of Oral Histology and Developmental Biology, School of Dentistry, Seoul National University, Seoul, South Korea; ^2^Regenerative Dental Medicine R and D Center, HysensBio Co., Ltd., Seoul, South Korea

**Keywords:** CPNE7, autophagy, odontoblast process, post-mitotic long-lived cell, reactivation

## Abstract

Dentin, which composes most of the tooth structure, is formed by odontoblasts, long-lived post-mitotic cells maintained throughout the entire life of the tooth. In mature odontoblasts, however, cellular activity is significantly weakened. Therefore, it is important to augment the cellular activity of mature odontoblasts to regenerate physiological dentin; however, no molecule regulating the cellular activity of mature odontoblasts has yet been identified. Here, we suggest that copine-7 (CPNE7) can reactivate the lost functions of mature odontoblasts by inducing autophagy. CPNE7 was observed to elevate the expression of microtubule-associated protein light chain 3-II (LC3-II), an autophagy marker, and autophagosome formation in the pre-odontoblast and mature odontoblast stages of human dental pulp cells. CPNE7-induced autophagy upregulated DSP and DMP-1, odontoblast differentiation and mineralization markers, and augmented dentin formation in mature odontoblasts. Furthermore, CPNE7 also upregulated NESTIN and TAU, which are expressed in the physiological odontoblast process, and stimulated the elongation of the odontoblast process by inducing autophagy. Moreover, lipofuscin, which progressively accumulates in long-lived post-mitotic cells and hinders their proper functions, was observed to be removed in recombinant CPNE7-treated mature odontoblasts. Thus, CPNE7-induced autophagy reactivated the function of mature odontoblasts and promoted the formation of physiological dentin *in vivo*. On the other hand, the well-known autophagy inducer, rapamycin, promoted odontoblast differentiation in pre-odontoblasts but did not properly reactivate the function of mature odontoblasts. These findings provide evidence that CPNE7 functionally reactivates mature odontoblasts and introduce its potential for dentinal loss-targeted clinical applications.

## Introduction

Teeth are hard, mineral-rich structures that serve to masticate food (Brudevold et al., 1960). Teeth also serve as a mineralized outer barrier that protect the inner dental pulp from external stimuli and damage (Craig and Peyton, 1958). Dentin makes up more than 70% of the entire tooth structure, and it forms a bridge between the dental pulp and the enamel through a dentinal tubule structure (Marshall, 1993). Some animals can replace their teeth throughout their lives (van Nievelt and Smith, 2005). However, humans have only two sets of teeth: deciduous teeth, which generally loosen and fall out prior to adulthood, and permanent teeth, which replace the deciduous teeth and stay in place for the remaining lifespan (Kim et al., 2014). Thus, severe injury to the permanent teeth is irreversible and results in extraction since dentin forming odontoblasts cannot be normally replaced in humans.

Odontoblasts are ectomesenchyme-derived post-mitotic cells that form a significant layer in the dentin-pulp complex. As they differentiate from the dental mesenchyme, odontoblasts (pre-odontoblasts) become highly polarized and form a cytoplasmic elongation called the *odontoblast process*. Leaving their processes in the dentinal tubules, odontoblasts (secretory odontoblasts) retreat from the dentin and actively secrete organic dentin matrix that is then progressively mineralized (Couve, 1986). However, when physiologic dentin is completed, active secretory odontoblasts gradually transform into a terminally differentiated stage (mature odontoblasts) with debilitated cellular function and responsiveness. Mature odontoblast’s longevity is sustained by elaborate autophagic activity that regulates organelle and protein renewal. However, progressive dysfunction of that system can debilitate the activity of mature odontoblasts and eventually impair their dentin maintenance capacity (Couve et al., 2013).

Copine-7 (CPNE7), a dental epithelium-derived factor, has been reported to promote odontogenic differentiation and physiological dentin formation (Oh et al., 2015). This secreted factor from the pre-ameloblasts, binds to nucleolin on the cell surface of pre-odontoblasts and translocates to the nucleus, where it regulates dentin sialophosphoprotein (*Dspp*) expression (Seo et al., 2017; Park et al., 2019). Of the numerous odontoblast differentiation-promoting factors reported so far, non-collagenous dentin matrix proteins (Duque et al., 2006; Kim, 2017) and CPNE7 (Choung et al., 2016; Lee et al., 2020) have been shown to form the physiological tubular dentin that contains the dentinal tubules that hold the odontoblast processes. However, the precise mechanism of CPNE7-mediated physiologic tubular dentin regeneration in adult dentin has not yet been elucidated. In the process of CPNE7-mediated physiological dentin regeneration, CPNE7 could augment the functional quality of mature odontoblasts and promote the elongation of their cellular processes into newly formed dentinal tubules via cellular remodeling. Considering the connections between microtubules, which contribute to odontoblast processes (Nishikawa and Kitamura, 1987), and autophagy, along with autophagy defect leading to impaired dentin (Park et al., 2020), the effects suggest a possible correlation between CPNE7 and autophagy in mature odontoblast and their potential to restore the cell’s functional and physiological activity.

In this study, we identify the association between CPNE7 and autophagy in mature odontoblasts and its effect in functional restoring. We hypothesized that CPNE7 may be connected with autophagy-mediated odontoblast process elongation and physiological dentin formation. Specifically, we aimed to evaluate (1) the involvement of autophagy in CPNE7-mediated odontoblast process elongation and mineralization *in vitro*, (2) the ramifications of CPNE7 in calcified tissue formation using subcutaneous transplantation *ex vivo*, and (3) the possible application of CPNE7-mediated physiological dentin formation using a mouse dentin exposure model *in vivo*.

## Results

### CPNE7 Induces Autophagic Activity in Both Pre-odontoblasts and Mature Odontoblasts

**FIGURE 1:**
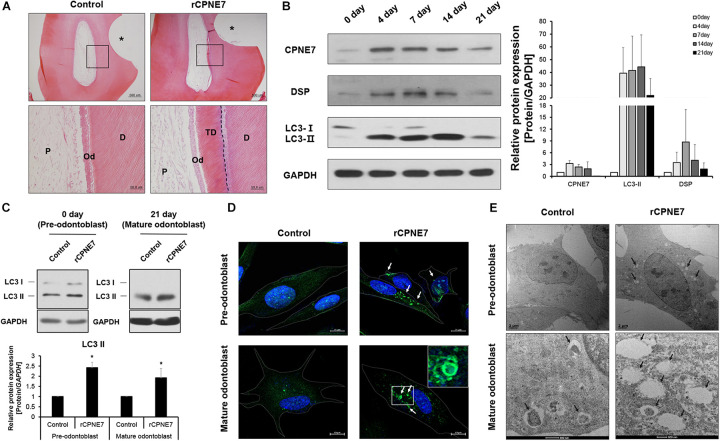
CPNE7 induces autophagic activity in both pre-odontoblasts and mature odontoblasts. **(A)** Histological analysis of beagle dog pre-molar defect model. Scale bars = 500 μm. The asterisk shows the tooth defect area. Boxed areas are shown at higher magnification. Scale bars = 50 μm. The dotted line shows the boundary of tubular dentin newly formed from native dentin. **(B)** Western blot analyzing the protein expression patterns of CPNE7, LC3, and DSP in primary human dental pulp cells (hDPCs) during odontogenic differentiation. **(C–E)** Autophagic activity was analyzed in control and rCPNE7-treated hDPCs at the pre-odontoblast stage (day 0) and the mature odontoblast stage (day 21). **(C)** LC3 II protein levels were evaluated by western blotting. **(D)** Representative immunofluorescence images of LC3 (green dot; white arrows). DAPI (blue) counterstaining indicates the nucleus. Scale bars = 10 μm. Boxed areas are shown at higher magnification. **(E)** Autophagic vacuoles (black arrows) were observed by transmission electron microscopy. Scale bars = 500 nm. hDPCs were treated with rCPNE7 (100ng/ml) for 24 h. CPNE7, copine-7; rCPNE7, recombinant CPNE7; P, pulp; Od, odontoblast; D, dentin; TD, tubular dentin; LC3, microtubule-associated protein 1A/1B-light chain 3; DSP, dentin sialoprotein. Significant differences are shown with asterisks. *P < 0.05.

Our previous studies showed that CPNE7 induces odontoblastic differentiation of human dental pulp cells (hDPCs) *in vitro* and promotes dentin formation *ex vivo* (Lee et al., 2011; Oh et al., 2015). Here, we investigated the role of CPNE7 in the mature odontoblasts of beagle dogs. Beagle dog pre-molar defect models were created by exposing the dentinal tubules through drilling. The prepared defects were divided into 2 groups: the non-treated control group and the recombinant human CPNE7 (rCPNE7)-treated group. The exposed dentin of the 2 groups was filled with glass ionomer cement (GI cement) after topical treatment. There was no change in the control group, but tubular dentin containing odontoblast processes was observed at the defect sites of the rCPNE7-treated group (Figure 1A). Those findings suggest that CPNE7 may augment their cellular function and result in physiological tubular dentin regeneration, despite mature odontoblast’s low capacity to react to external stimuli.

Next, we tried to define the approximate period when an odontoblast became terminally differentiated, or “mature” *in vitro*. hDPCs were cultured for 21 days in an odontoblast differentiation medium. Afterward, the expression patterns of CPNE7 and a representative odontoblast differentiation and mineralization marker, dentin sialoprotein (DSP), were analyzed. CPNE7 expression increased from days 0 to 7 (early stage of differentiation) and then progressively decreased until day 21 (late stage of differentiation). DSP showed a similar expression pattern, with its expression significantly weakening around day 21 (Figure 1B). Therefore, we set day 21 as the time at which odontoblasts reached a mature state with debilitated cellular activity and responsiveness.

A previous study demonstrated that autophagy is involved in odontoblast differentiation and tooth morphogenesis during tooth development (Park et al., 2020). To determine whether CPNE7 affects autophagic activity in odontoblasts, we identified changes in the levels of microtubule-associated protein light chain 3-II (LC3-II), a widely used autophagy marker, in rCPNE7-treated hDPCs. Upon the induction of autophagy, a cytosolic form of LC3 (LC3-I) conjugates to phosphatidylethanolamine to form an LC3-phospholipid conjugate (LC3-II), which is localized to autophagosomes and autolysosomes. Therefore, changes in LC3 localization have been used to measure autophagy (Tanida et al., 2004, 2008). Interestingly, LC3-II showed similar expression patterns to CPNE7 in differentiating hDPCs: increased expression in the pre-odontoblast state and decreased expression in the mature odontoblast state (Figure 1B). In both the pre-odontoblast and mature odontoblast states, LC3-II was significantly upregulated in the CPNE7-treated group compared to the control (Figure 1C). Immunofluorescence testing of LC3 showed only a few autophagic vacuoles in the control group, whereas many more were detected in the rCPNE7-treated cells (Figure 1D). We used transmission electron microscopy (TEM) to detect the presence of any ultrastructural differences in cells between the control and rCPNE7-treated groups. Consistently, more autophagic vacuoles were detected in cells in the rCPNE7-treated group than in the control (Figure 1E). Those results suggest that CPNE7 induces autophagy in both the pre-odontoblast and mature odontoblast states.

### CPNE7 Promotes Odontoblast Differentiation and Dentin Formation by Inducing Autophagy in a Manner Different From That of Rapamycin

**FIGURE 2:**
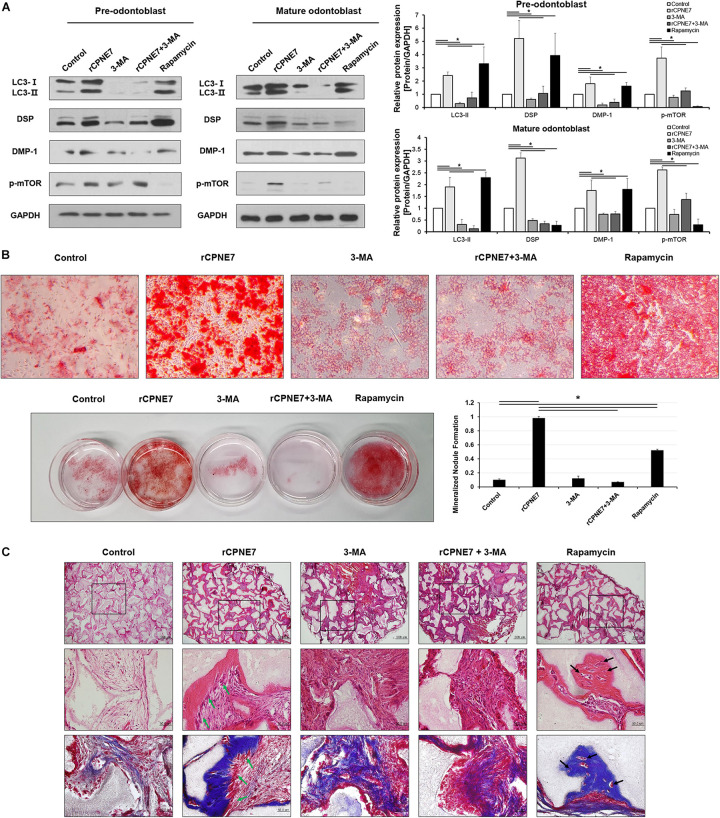
CPNE7 promotes odontoblast differentiation and dentin formation by inducing autophagy in mature odontoblasts. **(A)** Both stages of hDPCs were treated with rCPNE7 (100 ng/ml), 3-MA (5 μM), rCPNE7 + 3-MA, or rapamycin (10 nM) for 24 h. LC3 II, DSP, DMP-1, and p-mTOR protein levels were evaluated by western blot analysis. **(B)** Effects of rCPNE7, 3-MA, rCPNE7 + 3MA, and rapamycin on the mineralized nodule formation of mature hDPCs *in vitro*, as analyzed by alizarin red S staining. **(C)** Ectopic transplantation of mature odontoblasts treated with control (PBS), rCPNE7, 3-MA, rCPNE7 + 3-MA, or rapamycin and HA/TCP was histologically analyzed through H&E and Masson’s trichrome staining. Scale bars = 500 μm. Boxed areas are shown at higher magnification. Scale bars = 50 μm. Dentin-pulp-like structures (green arrows) were observed in the rCPNE7 groups. Cells entrapped in mineralized tissue (black arrows) were observed in the rapamycin groups. 3-MA, 3-methyladenine; DMP-1, dentin matrix protein 1; p-mTOR, phospho-mammalian target of rapamycin; HA/TCP, hydroxyapatite/tricalcium phosphate. Significant differences are shown with asterisks. *P < 0.05.

We next investigated how CPNE7 functions by inducing autophagy at each stage of odontoblast differentiation (pre-odontoblast and mature odontoblast). After treating both differentiation states with rCPNE7 for 24 h, we evaluated the expression levels of odontoblast differentiation and mineralization markers, DSP and DMP-1 proteins, respectively. Interestingly, rCPNE7 treatment significantly increased the expression levels of the odontoblast differentiation and dentin mineralization marker proteins in both states. To understand whether the CPNE7-induced autophagy regulated odontoblast differentiation and dentin mineralization, we used a PI3K inhibitor, 3-methlyadenine (3-MA), to block autophagic activity. When autophagic activity was inhibited in both differentiation states, the expression levels of LC3-II, DSP, and DMP-1 decreased. Interestingly, rCPNE7 treatment increased the expression levels of those proteins, but administering 3-MA after the rCPNE7 treatment counteracted the effects of rCPNE7 (Figure 2A). Therefore, CPNE7 promotes odontoblast differentiation and dentin mineralization by inducing autophagy. Next, we induced autophagy using rapamycin, a commonly used autophagy inducer, to compare its effects with those of CPNE7-induced autophagy in both differentiation states. Peculiarly, rapamycin-induced autophagy increased the levels of the odontoblast differentiation and mineralization marker proteins in the pre-odontoblast state, similar to CPNE7-induced autophagy; however, in mature odontoblasts, rapamycin-induced autophagy increased the expression of DMP-1 but decreased the expression of DSP (Figure 2A). Thus, CPNE7- and rapamycin-induced autophagy are not identical in their function in mature odontoblasts.

To further elucidate the pathway between CPNE7- and rapamycin-induced autophagy, we evaluated the effects of CPNE7 and rapamycin on phosphorylated mTOR (p-mTOR), the inhibition of which induces autophagy. p-mTOR decreased after treatment with rapamycin, whereas it increased in the CPNE7-treated group (Figure 2A), suggesting that CPNE7 and rapamycin induce autophagy through different pathways in mature odontoblasts. Following that expression analysis, we analyzed the dentin formation capacity of mature odontoblasts *in vitro* and *ex vivo*. To analyze the capacity of dentin formation, most studies have focused on the entire process of odontoblast differentiation, from the stem cell stage to the mature odontoblast stage. However, those methods make it difficult to determine the capacity of dentin formation of mature odontoblasts. In this study, we analyzed the *in vitro* mineralization capacity of mature odontoblasts using cells at specific differentiation stages. Mature odontoblasts treated with rCPNE7 showed significantly more mineralized nodule formation than the control. The rapamycin-treated group also showed more mineralized nodules than the control, but its effects were weaker than those seen in the rCPNE7 group. On the other hand, the number of mineralized nodules was significantly reduced when 3-MA was administered alone or with rCPNE7 (Figure 2B and Supplementary Figure 1). Therefore, CPNE7 enhances the mineralization potential of mature odontoblasts by inducing autophagy.

We next tested the role of CPNE7-induced autophagy in enhancing the cellular activity and mineralization capacity of mature odontoblasts *ex vivo*. hDPCs differentiated until day 21 were transplanted into the subcutaneous tissues of immunocompromised mice along with hydroxyapatite/tricalcium phosphate (HA/TCP) in five different conditions: PBS treatment (control), rCPNE7-treatment (rCPNE7), 3-MA treatment (3-MA), rCPNE7 and 3-MA treatment (rCPNE7 + 3-MA), and rapamycin-treatment (rapamycin). After 6 weeks, the tissues newly formed around the HA/TCP were observed. Similar to our *in vitro* results, cellular function and physiological activity were debilitated in mature odontoblasts, and little hard tissue formation was observed in the control group (Figures 2B,C). The rCPNE7 group showed significantly more dentin-pulp-like tissue formation than the control, and the 3-MA group showed abnormal cell morphology and no hard tissue formation. The addition of 3-MA offset the effects of rCPNE7 because little hard tissue formation was observed in the rCPNE7 + 3-MA group. In the rapamycin group, a smaller amount of hard tissue was formed than in the rCPNE7 group, and irregular hard tissue in which cells were entrapped was observed. Masson’s trichrome staining further confirmed that CPNE7 enhanced the mineralization capacity of mature odontoblasts and that autophagy inhibition impaired it *ex vivo* (Figure 2C). Thus, CPNE7 fosters the cellular activity and mineralization potential of mature odontoblasts in a way that depends on autophagy.

### CPNE7 Stimulates Odontoblast Process Elongation

**FIGURE 3:**
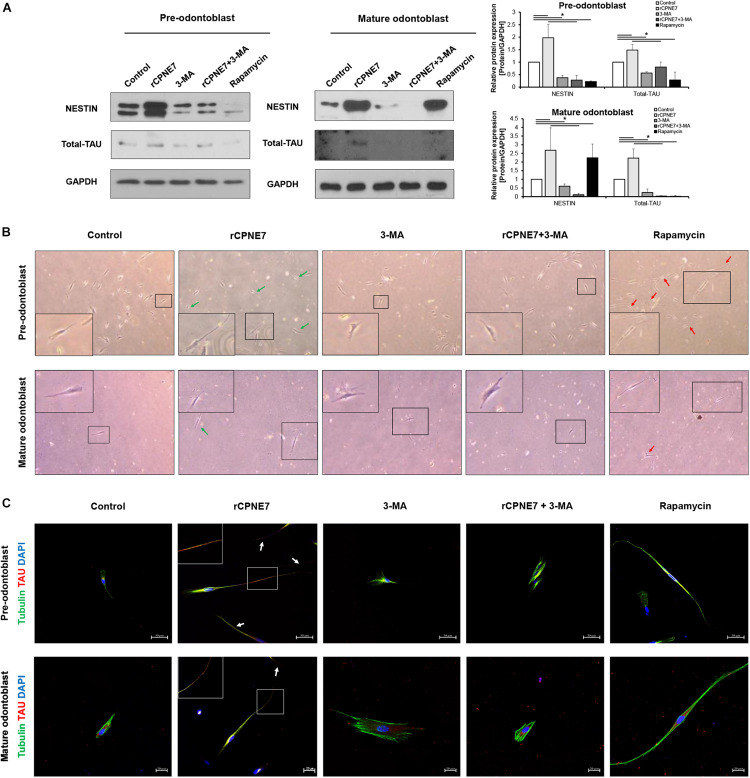
CPNE7 stimulates odontoblast process elongation. **(A)** Both stages of hDPCs were treated with rCPNE7, 3-MA, rCPNE7 + 3-MA, or rapamycin for 24 h. NESTIN and total-TAU protein levels were evaluated by western blot analysis. **(B,C)** The morphology of hDPCs was analyzed in each group 7 days after treatment. Boxed areas are shown at higher magnification. **(B)** Cells were observed under an optical microscope (100 x). Odontoblasts with a unidirectional odontoblast process-like structure (green arrows) were observed in the rCPNE7 group. Multidirectional odontoblast process-like structures were observed (red arrowheads) in the rapamycin group. **(C)** Representative immunofluorescence images of TUBULIN (green) and TAU (red) in each group of treated cells. TUBULIN and TAU expression was co-localized in the odontoblast process-like structures in the rCPNE7 group (arrows). Scale bars = 50 μm and 20 μm. Significant differences are shown with asterisks. *P < 0.05.

After demonstrating that CPNE7 induces tubular dentin formation (Figure 1A), we explored whether CPNE7 promotes odontoblast process elongation by examining changes in the expression levels of microtubule-associated proteins, TAU and NESTIN, in both differentiation states. TAU and NESTIN are terminally differentiated odontoblast marker proteins that participate in odontoblast morphogenesis by regulating microtubule organization and intermediate filaments, respectively (Miyazaki et al., 2015). rCPNE7 treatment remarkably increased the expression of both proteins compared to the control group. In contrast, the groups treated with 3-MA, alone or with rCPNE7, showed decreased expression levels in both differentiation states. Interestingly, the rapamycin group had significantly decreased TAU levels compared to the rCPNE7 group in both differentiation states, whereas the NESTIN levels decreased after rapamycin treatment in pre-odontoblasts and increased in mature odontoblasts, compared with the control (Figure 3A). Overall, the rapamycin group did not show obvious associations with cytoskeletal related markers, unlike the rCPNE7 group, which showed increases in both markers. Following that expression analysis, we conducted a cell morphological analysis to examine whether CPNE7 promotes physiological odontoblast process elongation in both differentiation stages. Under a light microscope, unidirectional odontoblast process-like structures were visible in the control groups of pre-odontoblasts and mature odontoblasts cultured in an odontoblast differentiation medium for 7 days. Morphologically, as odontoblasts differentiate, the cell bodies retreat by elongating their single cellular processes, known as the *odontoblast process* (Arana-Chavez and Massa, 2004). In both differentiation stages, the rCPNE7 group showed significantly longer unidirectional odontoblast process-like structures than the control group (Supplementary Figure 2), and the rapamycin group showed multidirectional cellular processes that differed from the typical physiological odontoblast morphology. Those cell morphologies were not observed in the autophagy-inhibited groups (Figure 3B). We further evaluated each of the cell morphologies by performing a co-immunofluorescence assay for TAU and TUBULIN. Similar to the western blotting, the rCPNE7 group showed elevated TAU expression in both stages of odontoblasts. Interestingly, in the rCPNE7 group, TAU was strongly co-localized with TUBULIN at the tip of the unidirectional odontoblast process-like structure, whereas co-localization was rarely seen in the other groups (Figure 3C). Thus, CPNE7 upregulates the expression of TAU and might be involved in cellular remodeling, and it eventually promotes odontoblast process elongation in both differentiation states.

### CPNE7 Removes Lipofuscin From Mature Odontoblasts

**FIGURE 4:**
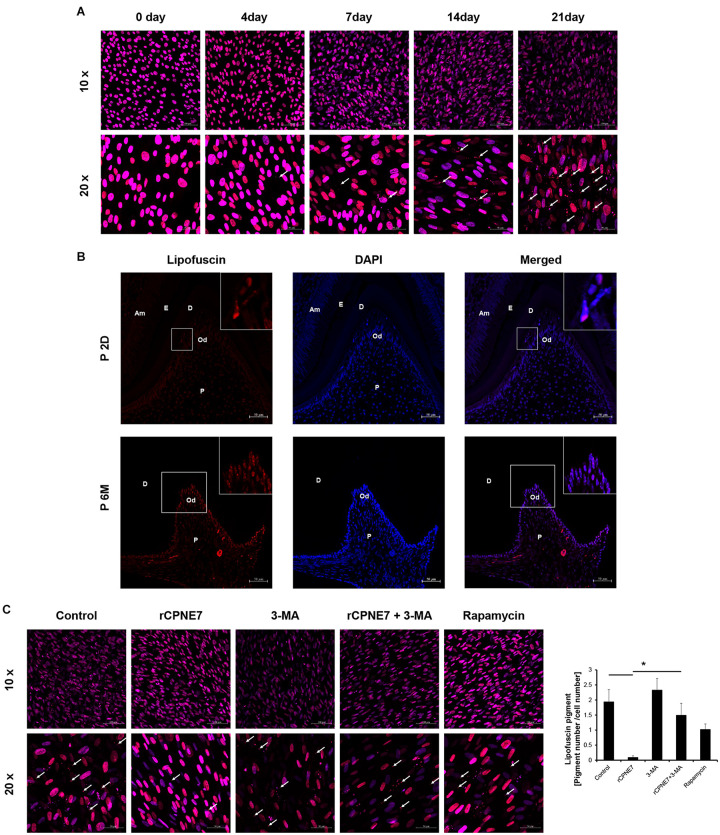
CPNE7 removed lipofuscin in mature odontoblasts. **(A)** Lipofuscin (red dots; white arrows) accumulation was observed during the differentiation of hDPCs. **(B)** Lipofuscin pigments were observed in mature odontoblasts on postnatal day 2 (P2D) and at postnatal 6 months (P6M). Scale bars = 50 μm. Boxed areas are shown at higher magnification. **(C)** Lipofuscin (white arrows) was analyzed in the control, rCPNE7, rCPNE7 + 3-MA, and rapamycin groups of mature odontoblasts. Scale bars = 10x: 100 μm and 20x: 50 μm. Am, ameloblast; E, enamel; P, pulp; Od, odontoblast; D, dentin. Significant differences are shown with asterisks. *P < 0.05.

Lipofuscin is a fluorescent pigment that accumulates with aging in the lysosomal compartment of post-mitotic cells such as odontoblasts and neurons (Rezzani et al., 2012). Because of

the correlation between its accumulation and aging, lipofuscin has long been reported to be pathogenic (Terman and Brunk, 2004). Confocal microscope was used to check the accumulation of lipofuscin pigment following odontoblast differentiation *in vitro* (Figure 4A). We also identified the extent of lipofuscin accumulation in odontoblasts *in vivo* in post-natal day 2 mice (young mice) and post-natal 6-month-old mice (aged mice). Consistent with previous results, lipofuscin pigments were rarely observed in the early differentiating odontoblasts of young mice, and they amassed in the mature odontoblasts of the aged mice (Figure 4B). Because we demonstrated that lipofuscin actually accumulates following differentiation and that the cellular function and physiological activity of mature odontoblasts are debilitated, our next question was whether CPNE7-induced autophagy could eliminate lipofuscin. Interestingly, treating mature odontoblasts with rCPNE7 decreased the accumulation of lipofuscin compared with the control and autophagy-inhibited groups (Supplementary Figure 3). In the rapamycin-treated group, however, lipofuscins were observed to a greater extent than in the rCPNE7-treated group in mature odontoblasts (Figure 4C). These results suggest that CPNE7-induced autophagy could cause long-lived post-mitotic odontoblasts to revert to the cellular function and physiological activity of their active state, perhaps by removing lipofuscin.

### CPNE7 Promotes Physiological Dentin Formation *in vivo*

**FIGURE 5:**
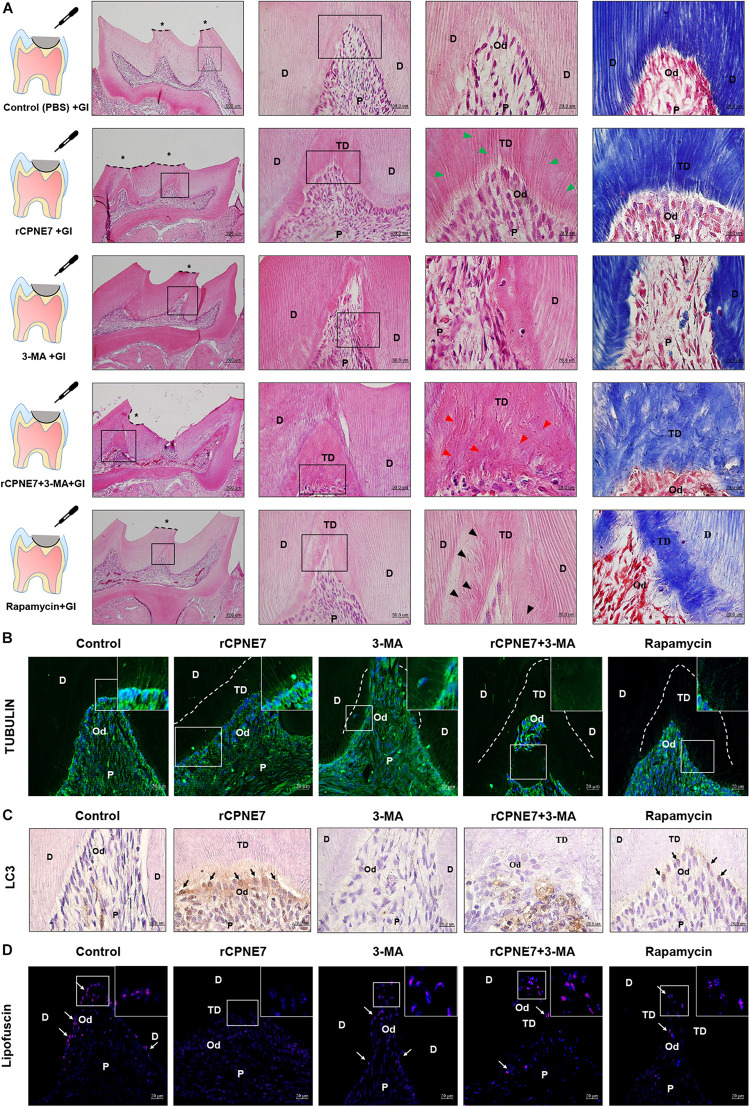
CPNE7 promotes the formation of dentin *in vivo*. **(A)** Schematic diagrams of the mouse molar defect model. The defect areas were covered with glass ionomer (GI) cement after topical treatment: control (PBS + GI), rCPNE7 (rCPNE7 + GI), 3-MA (3-MA + GI), rCPNE7 and 3-MA (rCPNE7 + 3-MA + GI), or rapamycin (rapamycin + GI). Histologic analysis of the defect areas were performed 4 weeks later using H&E and Masson’s trichrome staining. Scale bars = 200 μm. Boxed areas are shown at higher magnification. Scale bars = 50 μm and 20 μm. Odontoblast process-like structures (green arrowheads) were observed in the rCPNE7 + GI group. Few odontoblast process-like structures (red arrowheads) were observed in the rCPNE7 + 3-MA + GI group. Irregular and entangled cellular processes (arrowheads) were observed in the rapamycin + GI group. **(B)** Representative immunofluorescence images of tubulin (green). Dotted line shows the boundary of tubular dentin newly formed from native dentin. **(C)** LC3 expression (black arrow) was detected by immunohistochemistry. **(D)** Lipofuscin pigments (white arrows) were observed in odontoblasts at 488 nm. Scale bars = 20 μm. Boxed areas are shown at higher magnification. P, pulp; Od, odontoblast; D, dentin; TD, tubular dentin.

A mouse molar defect model with exposed dentinal tubules was created using drilling to evaluate the role of CPNE7-induced autophagy in mature odontoblasts and physiological dentin formation *in vivo*. The defects were topically treated with PBS (control), rCPNE7, 3-MA, rCPNE7 + 3-MA, or rapamycin. The exposed dentin areas of the five groups were filled with GI cement after the topical treatment, and the reactions of the mature odontoblasts were histologically analyzed 4 weeks later. In the control group, no changes were observed. However, in the rCPNE7 group, a large amount of tubular dentin formation was observed below the dentinal defect site. In the 3-MA group, we observed a hard tissue without dentinal tubule structure entrapping a small number of cells. Interestingly, in the group that treated CPNE7 and 3-MA together, dentin was formed, but the odontoblast process was hardly seen. Similar to our *in vitro* cell morphological analysis, the rapamycin-treated odontoblasts formed dentin with irregular and entangled cellular processes *in vivo* (Figures 5A,B). Furthermore, compared to the control group, LC3-positive odontoblasts and clearly removed lipofuscin were detected below the newly formed tubular dentin in the rCPNE7 group. However, no LC3-postive odontoblasts and large amounts of lipofuscins were observed in autophagy-inhibited groups. Although some LC3 positive cells were detected, the lipofuscin pigments were still observed in Rapamycin-treated group *in vivo*, similar to the results of *in vitro* experiments (Figures 5C,D). Thus, CPNE7-induced autophagy might reactivate the debilitated physiological activity of mature odontoblasts and promote the formation of physiological dentin.

## Discussion

Autophagy is a highly conserved, lysosome-mediated, self-degradation mechanism that allows cells and tissues to cope with stimuli such as starvation, infection, and pharmacological agents. In physiological conditions, this cellular system finely regulates processes indispensable for development, differentiation, and survival (Rabinowitz and White, 2010). The inhibition of autophagy in mouse tooth germs contributes to the loss of odontoblast polarization and poor differentiation, which eventually lead to highly disrupted tooth morphology (Park et al., 2020). Thus, the proper modulation of autophagic activity is essential for odontoblast differentiation and physiological dentin formation. Moreover, mature odontoblasts, whose secretory machinery and autophagic activity are reduced, do not react properly to external stimuli (Couve, 1986). In this study, the cellular activity and dentin formation capability of mature odontoblasts became debilitated as autophagic activity decreased. If autophagic activity could be increased in mature odontoblasts, their cellular activity and dentin formation ability might revert to that of active secretory odontoblasts.

In this study, we found that CPNE7-induced autophagy increased the expression of odontoblast differentiation and mineralization marker proteins in both pre-odontoblasts and mature odontoblasts. The effects of rapamycin treatment on pre-odontoblasts and mature odontoblasts differed from those of CPNE7-induced autophagy. Rapamycin-induced autophagy promoted odontoblast differentiation by upregulating DSP and DMP-1 in pre-odontoblasts, similar to CPNE7-induced autophagy. However, in mature odontoblasts, the DSP expression level in the rapamycin group decreased, unlike the results from CPNE7-induced autophagy. There are two types of autophagy-induced pathway: mTOR-dependent and independent. As a general autophagy inducer and p-mTOR inhibitor, Rapamycin was used in our experiment. Indeed, the expression of p-mTOR and LC3-II was decreased and increased respectively in rapamycin-treated both differentiation stages of odontoblasts. This result suggests that Rapamycin-induced autophagy is an mTOR-dependent autophagy pathway. However, when CPNE7 was treated in both stages of odontoblasts, p-mTOR and LC3-II were increased, which suggests CPNE7 could induce autophagy through mTOR-independent pathway, and this CPNE7-induced autophagy may have different functions than the mTOR-dependent autophagy pathway.

Odontoblast processes and dentinal tubules are typical morphological characteristics of the physiological dentin-pulp complex. During dentin formation, the odontoblast process is elongated gradually as a direct extension of the cell body, along with continuous remodeling of the plasma membrane. The odontoblast process sequentially secretes matrix vesicles and is eventually surrounded by a calcified matrix, the dentinal tubule. The odontoblast process consists mostly of a cytoskeleton, including microtubules. It has been suggested that the microtubules not only serve a cytoskeletal function but also play a role in the transport of release of matrix vesicles (Lee et al., 2014). In any case, odontoblast process formation and elongation are essential for physiological dentin formation. In this study, we observed that long unidirectional odontoblast processes were formed in response to CPNE7-mediated autophagy in both stages of odontoblasts. On the other hand, rapamycin treatment of both stages of odontoblasts produced multidirectional cellular processes. Furthermore, increased expression levels of TAU were observed after CPNE7 treatment, while they decreased after rapamycin treatment. TAU is a neuronal phosphoprotein that modulates microtubule dynamics and assembly and has various neuronal developmental functions. Moreover, TAU and TUBULIN are co-expressed in odontoblasts upon the onset of dentinogenesis, and they are localized mainly in the apical regions of the cell body and cell processes, similarly to the expression patterns seen for NESTIN in mature odontoblasts (Miyazaki et al., 2015). Our confocal microscopy results confirm that TAU expression increased and was co-localized with TUBLIN in rCPNE7-treated unidirectional odontoblast processes. The co-localization of TAU and TUBLIN was not observed in the rapamycin-treated multidirectional cellular processes. Thus, only CPNE7-induced autophagy can promote the formation and elongation of physiological odontoblast processes. The reason behind the multidirectional cellular process after the rapamycin treatment requires further research.

Mature or terminally differentiated cells that no longer undergo mitosis, including odontoblasts, neurons, and cardiomyocytes, are called *long-lived post-mitotic cells* (Terman et al., 2010). Long-lived post-mitotic cells, including odontoblasts, amass lipofuscin, which is regarded as a reliable biomarker of aging cells. The main cause of lipofuscin formation is the incomplete lysosomal degradation of damaged mitochondria. Autophagic activity fails to function properly as lipofuscin accumulates in mature odontoblasts, which eventually compromises cellular activity (Couve et al., 2013). In this study, in the autophagy-inhibited groups (3-MA and rCPNE7 + 3-MA), a large amount of lipofuscin was observed in mature odontoblasts similarly to that of the control group. Interestingly, when CPNE7 was administered to mature odontoblasts *in vitro* and *in vivo*, most of the lipofuscin was removed, whereas rapamycin treatment did not clearly eliminate lipofuscin. Thus, CPNE7-induced autophagy could cause mature odontoblasts to revert to the cellular function and physiological activity of active secretory odontoblasts by removing lipofuscin. The precise mechanism by which CPNE7-induced autophagy eliminates lipofuscin from mature odontoblasts requires further study.

**FIGURE 6:**
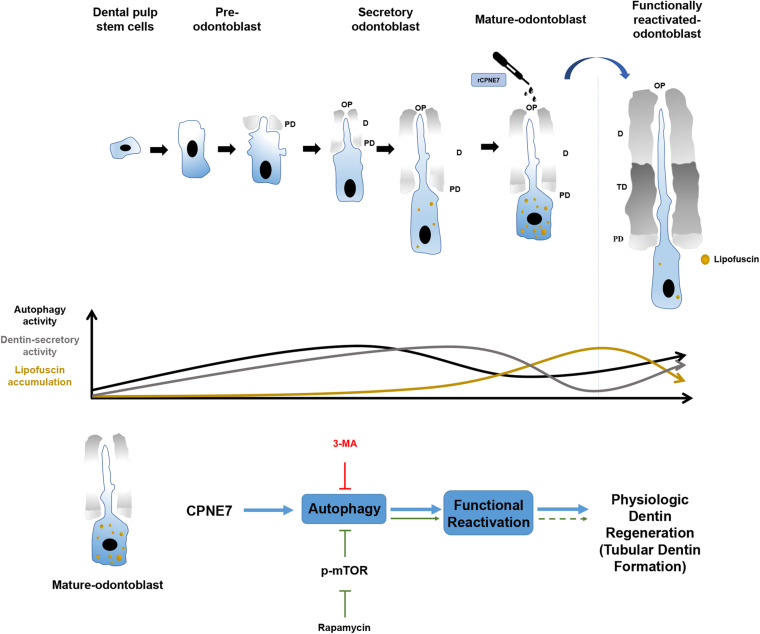
Schematic illustration of the effects of CPNE7 in mature odontoblasts. Dental pulp stem cells have the potential to differentiate into pre-odontoblasts, secretory odontoblasts, and mature odontoblasts. Physiologically, the cellular activities of odontoblasts, such as dentin secretion and process elongation, increase gradually during the early differentiation stage, similar to the increasing autophagy activity. Both the cellular activities of odontoblasts and autophagic activity decrease steadily in mature odontoblasts. Moreover, lipofuscin, an aging pigment, progressively accumulates in mature odontoblasts. Rapamycin-induced autophagy could not properly restore the physiological activity of mature odontoblasts. However, CPNE7-induced autophagy functionally reactivated mature odontoblasts and produced physiological dentin formation.

In this study, rCPNE7 treatment of exposed dentin generated a massive area of tubular dentin containing physiological odontoblast processes, whereas rapamycin treatment produced a narrow area of dentin containing irregular and entangled odontoblast processes. Furthermore, the mature odontoblasts in the rCPNE7-treated group contained less lipofuscin under the newly formed dentin than the control group. The most interesting finding was that when CPNE7 and 3-MA were administered to the defective dentin together, dentin formation was disrupted and showed few odontoblast processes. Therefore, CPNE7-induced autophagy is an innovative process for reactivating the cellular activity of and removing lipofuscin from mature odontoblasts. That process promotes odontoblast process elongation and dentin formation, which eventually leads to physiological dentin regeneration (Figure 6).

In conclusion, the findings of the present study hint the potential application of CPNE7-mediated autophagy in mature odontoblast’s cellular activity reactivation and physiological dentin regeneration in treating dentinal defects. More specifically, CPNE7 was seen to promote physiological odontoblast process elongation and dentin formation. In addition, while exploring CPNE7-induced autophagy, we observed lipofuscin elimination and the possibility of reactivating long-lived post mitotic cells, such as neuron cells and cardiomyocytes. On the top of its CPNE7’s potential application in dentin regeneration and dental pulp protection, this possibility suggests that CPNE7-induced autophagy may be a potential therapeutic material for neurodegenerative diseases. Therefore, further studies are needed to elucidate the effects of CPNE7-induced autophagy and whether it induces functional reactivation in other long-lived post mitotic cells.

## Materials and Methods

### Indirect Pulp Capping Model With Canine Teeth

All experiments using beagles were conducted under the guidelines provided by the Institutional Animal Care and Use Committee of Seoul National University (SNU-171020-5-2). The indirect pulp capping was performed as described previously (Lee et al., 2020). Three beagle dogs (aged 1-2 y) were examined throughout the experiment. Each group consisted of 2 premolars per animal, totaling 6 premolars. Briefly, after class V cavity preparation, cavities were either untreated or treated with a topical application of rCPNE7 protein (1 μg per tooth). The animals were sacrificed after 4 weeks of post-operation. The experimented beagle teeth were fixed at 4% PFA for 24 h. After fixation, the experimented teeth were decalcified in 10% formic acid (Georgia Chem, 64-18-6) over a month. For histological analysis, the decalcified tissues were embedded in paraffin. 5-μm serial sections were mounted on silanized slides and stored in air-tight cases at 4°C. For the histomorphometric analysis, samples were analyzed with H&E staining.

### Cell Culture

The experimental protocol for this study was approved by the Institutional Review Board (S-D20140007). All methods were performed in accordance with the relevant guidelines and regulations. Informed consent was obtained from all participants. Impacted human third molars were collected from the Seoul National University Dental Hospital (Seoul, South Korea). The human whole pulp cell isolation was executed as described previously (Jung et al., 2011). hDPCs were cultured in minimum essential medium α (Gibco BRL, 12571063) supplemented with 10% heat-inactivated fetal bovine serum (FBS; Gibco BRL, 16000044) and antibiotic-antimycotic (Gibco BRL, 15240062) at 37 °C in an atmosphere with 5% CO2. To induce the hDPCs into odontogenic differentiation, 80–90% confluent cells were cultured with 5% FBS, ascorbic acid (50 μg/Mℓ; VWR, 0143), and β-glycerophosphate (10 mM; Sigma-Aldrich, G9422) for up to 21 days. The cells were then treated with recombinant CPNE7 (rCPNE7, 100 ng/ml; Origene, TP306428), 3-methyladenine (3-MA, 5 mM; Sigma-Aldrich, M9281), or rapamycin (100 nM; Sigma-Aldrich, R8781) for 24 h and harvested. For the cell morphological analysis, differentiated and undifferentiated hDPCs were seeded into 35 mm confocal dishes (SPL, 101350) at a density of 1 × 10^3^ cells per dish. The cells were then treated with rCPNE7, 3-MA, or rapamycin for 7 days. An optical microscope (Olympus Co., CKX41) connected to a computer and charge-coupled device camera (IMT i-solution Inc., IMTcamCCD Pro2) was used to take images of the samples.

### Western Blot Analysis

Western blotting was performed as previously described (Seo et al., 2017). Affinity-purified rabbit polyclonal anti-CPNE7 and anti-DSP antibodies were produced as previously described (Lee et al., 2010; Lee et al., 2011). Anti-LC3 B polyclonal antibody (Abcam, ab48394), anti-TAU monoclonal antibody (TAU-5; Thermo Fisher Scientific, AHB0042), anti-phospho-mTOR (mechanistic target of rapamycin kinase) polyclonal antibody (Cell Signaling Technology, 5536), anti-DMP-1 (dentin matrix protein 1) polyclonal antibody (Abcam, ab103203), anti-NESTIN monoclonal antibody (10C2; Thermo Fisher Scientific, MA1-110), and anti-GAPDH monoclonal antibody (GA1R; Thermo Fisher Scientific, MA5-15738) were purchased from the respective companies. Protein loading was assessed by the expression of GAPDH. All reactions were performed in triplicate. Semi-quantitative analysis were performed using ImageJ software (National Institute of Health).

### Immunocytochemistry and Immunofluorescence

Human dental pulp cells fixed in 4% paraformaldehyde (PFA; T&I, BFA-9020) and deparaffinized tissue sections were treated with phosphate-buffered saline (PBS) containing 0.5% Triton X-100 (Amresco, 0694) for permeabilization. After being washed and blocked, the cells were incubated for 1 h with LC3B antibody (1:100) or α-TUBULIN (1:100, YOL1/34; Santa Cruz Biotechnology, sc-53030) antibody and TAU antibody (1:50) in blocking buffer (PBS and 2% bovine serum albumin (BSA; Gibco BRL, 30063-572)). Tissue sections were incubated with α-TUBULIN antibody (1:200) overnight at 4°C. Subsequently, fluorescein isothiocyanate (FITC)-conjugated goat anti-rabbit IgG antibody (1:200; Thermo Fisher Scientific, F2765), Alexa Fluor 488-conjugated goat anti-rat IgG antibody (1:200; Thermo Fisher Scientific, A11006), or Cy3-conjugated goat anti-mouse IgG antibody (1:200; Merck Millipore, AP124C) was applied. After being washed, the chromosomal DNA in the nucleus was stained with 4’,6-diamidino-2-phenylindole (DAPI) during the mounting procedure (Vector Labs, H-5000). Samples were visualized using confocal laser scanning microscopy (Carl Zeiss, LSM 800).

### TEM Analysis

Cells were harvested with trypsin and ethylenediaminetetraacetic acid (EDTA) and fixed with 2.5% glutaraldehyde (GA; Sigma-Aldrich, G7651) in PBS. They were then post-fixed in buffered 1% osmium tetroxide, embedded and fixed a second time in 2.5% GA, cut into 1 mm sections, and dehydrated through a graded ethanol series and propylene oxide prior to microwave infiltration of 1:1 Spurr/Epon resin. The polymerized blocks were sectioned on an ultramicrotome (Leica, EM UC6), and 70-nm sections were re-mounted on 100-mesh grids and stained with uranyl acetate and Reynolds lead citrate. Images were acquired with a JEM-1400 Flash (JEOL).

### Alizarin Red Staining for Mineralized Matrix

hDPCs were cultured with an odontogenic induction medium for 21 days. On day 21, the cells were seeded into 35 mm culture dishes at a density of 1 × 10^5^ cells per dish. The cells were then treated with rCPNE7, 3-MA, rCPNE7 + 3-MA, or rapamycin and cultured in odontogenic induction medium for 7 more days. After 7 days, cells were fixed with 4% PFA overnight at 4°C and stained with 40 mM alizarin red S (Sigma-Aldrich, A5533), pH 4.2, for 30 min at room temperature. To quantify the mineralized matrix in culture, alizarin red stain was eluted using 0.5 mL of 5% sodium dodecyl sulfate (Amresco, 0227) in 0.5 N HCl solution and shaken for 30 min; the absorbance of the eluted dye was measured at 405 nm.

### Ectopic Transplantation *in vivo* and Histological Analysis

Primary cultured hDPCs were differentiated for 21 days *in vitro*. Differentiated cells (2 × 10^6^) were mixed with 100 mg of hydroxyapatite/tricalcium phosphate (HA/TCP) ceramic powder (Zimmer Biomet, 00110900612) alone or with either rCPNE7 (1 μg), 3-MA (40 mM), rCPNE7 + 3-MA, or rapamycin in a 0.5% fibrin gel. The cells were then transplanted subcutaneously into immunocompromised mice (NIH-bg- nu-xid; Harlan Laboratories) for 6 weeks. For the histomorphometric analysis of the newly formed mineralized tissue, samples were harvested and fixed in 4% PFA, decalcified in 10% EDTA (pH 7.4; Georgia Chem, ED2041), embedded in paraffin, and stained with hematoxylin and eosin (H&E) and Masson’s trichrome (Polysciences Inc, 25088).

### H&E and Masson’s Trichrome Staining

Tissue sections were deparaffinized in xylene (DUKSAN Chemicals, UN1307) and rehydrated with ethanol (DUKSAN Chemicals, UN1170). For H&E staining, sections were stained with hematoxylin (Vector Labs, H-3401) for 4 min and eosin (T&I, BEY-9005) for 2 min. For Masson’s trichrome staining, sections were stained with Weigert’s hematoxylin for 10 min, 1% Biebrich-scarlet-acid fuchsin solution for 20 min, 5% phosphotungstic acid for 10 min, and 2.5% aniline blue solution for 20 min and then differentiated in 1% acetic acid for 1 min.

### Lipofuscin Analysis

Lipofuscin accumulation was characterized in tissue sections from coronal odontoblasts and primary cells labeled with DAPI. Lipofuscin deposits were visualized and analyzed under confocal laser scanning microscopy with a 488-nm laser line for excitation.

### Mouse Indirect Pulp Capping and Tissue Preparation

All experiments using mice followed protocols approved by the Institutional Animal Care and Use Committee of Seoul National University (SNU-171115-3-6). Fifteen mice (aged 1 M) were used for this experiment. The mice were anesthetized with a 2.5% 2-methyl-2-butanol (Avertin; Sigma, 240486) solution diluted with RNase-free water (T&I, BWA-8000). The oral cavity was opened with a mouth retractor to expose the molars. The maxillary first molars were cleaned with 0.5% chlorhexidine. Then, the center of the first molar was drilled using a carbide bur (FG1/4). The drilling stopped previously to pulp exposure. The cavity treatments were divided into 5 groups: (1) control (PBS + GI), (2) rCPNE7 (rCPNE7 + GI), (3) 3-MA (3-MA + GI), (4) rCPNE7 and 3-MA (rCPNE7 + 3-MA + GI), and (5) rapamycin (rapamycin + GI). The defect areas were covered with GI cement after the topical treatment appropriate to each group. Each group contained 2 first molars per animal, totaling 6 first molars per group. The animals were sacrificed 4 weeks post-operation. The mouse heads were fixed in 4% PFA in PBS for 24 h at 4°C. After fixation, the heads were decalcified in 10% EDTA (pH 7.4), embedded in paraffin, and cut for histological analysis. 4-μm serial sections were mounted on silanized slides and stored in air-tight cases at 4°C. All tissues were sectioned on the frontal plane. For the histomorphometric analysis, samples were analyzed with H&E and Masson’s trichrome staining or processed for immunohistochemistry.

### Immunohistochemistry

Sections were incubated overnight at 4°C with rabbit polyclonal LC3B antibody (1:200) in 2% BSA/PBS, pH 7.4. Negative control sections were incubated in 2% BSA/PBS. Sections were then incubated in 2% BSA/PBS mixed with biotin-labeled goat anti-rabbit immunoglobulin G (IgG) (1:200; Vector Labs, BP-9100) as the secondary antibody, washed, and incubated in avidin-biotin-peroxidase complex (Vector Labs, PK-6100). Peroxidase was revealed by incubation with methanol containing 3% H2O2 (JUNSEI, 7722-84-1). Signals were converted using a diaminobenzidine kit (Vector Labs, SK-4100). Nuclei were stained with hematoxylin.

### Statistical Analysis

All values are expressed as the mean ± SD of at least three independent experiments. Statistical significance between two groups was determined using Mann-Whitney U test. Differences were considered statistically significant at ^∗^*p* < 0.05.

## Data Availability Statement

The original contributions presented in the study are included in the article/Supplementary Material, further inquiries can be directed to the corresponding author.

## Ethics Statement

The animal study was reviewed and approved by the Institutional Animal Care and Use Committee of Seoul National University.

## Author Contributions

Y-HP, CS, Y-MS, and J-CP designed research. Y-HP, CS, Y-MS, and YL performed research. Y-HP, CS, and J-CP analyzed data. Y-HP, CS, AH, and J-CP wrote the manuscript. All authors contributed to manuscript revision, read, and approved the submitted version.

## Conflict of Interest

Y-HP and J-CP were employed by the company HysensBio Co., Ltd. J-CP is founder of HysensBio Co., Ltd. The remaining authors declare that the research was conducted in the absence of any commercial or financial relationships that could be construed as a potential conflict of interest.
