# Supplementary material for: CPNE7-Induced Autophagy Restores the Physiological Function of Mature Odontoblasts
Source: Front Cell Dev Biol. 2021 Apr 26;9:655498. doi: 10.3389/fcell.2021.655498 (PMC8107363; doi:10.3389/fcell.2021.655498)
Supplement: Supplementary file 1 [file Table_1.DOCX]

Supplementary Material

## Supplementary Figures


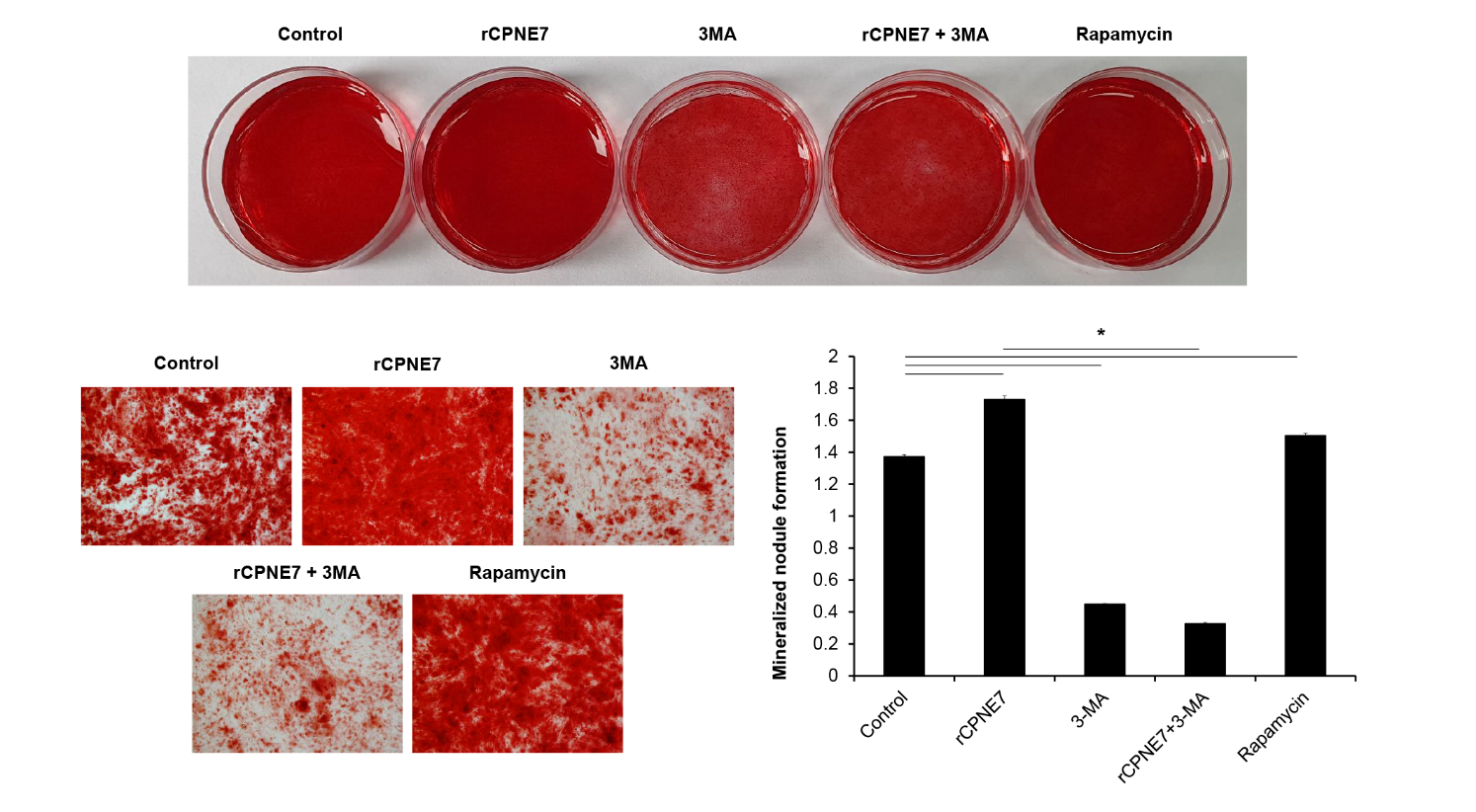


**Supplementary Figure 1.** CPNE7 promotes mineralized nodule formation by inducing autophagy in mature odontoblast. Effects of rCPNE7, 3-MA, rCPNE7+3MA, and rapamycin on the mineralized nodule formation from day 14 to day 21, as analyzed by alizarin red S staining. Significant differences are shown with asterisks. *P < 0.05.

**
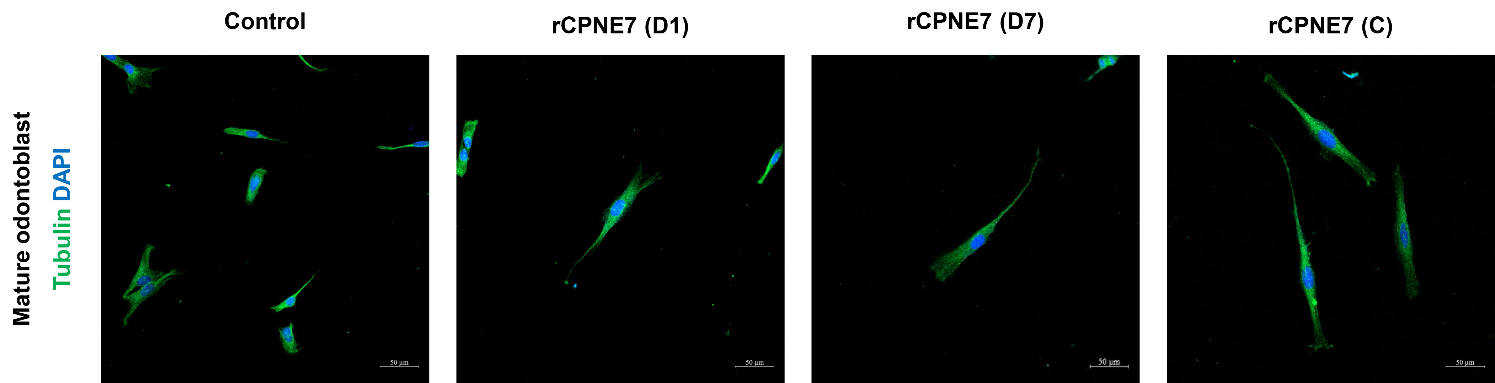
**

**Supplementary Figure 2.** CPNE7 stimulates odontoblast process elongation in mature odontoblast. hDPC were differentiated for 21 days with rCpne7 treatment for the following(indicated) period of time. At mature stage (D1; 20 – 21 days), secretory to mature stage (D7; 14 – 21 days), whole differentiation stage (C; continuously, 0 – 21 days) of odontoblast. Localization of TUBULIN (green) was observed by immunofluorescence. DAPI (blue) was counterstained to indicate the nucleus. Scale bars: 50 μm.

**
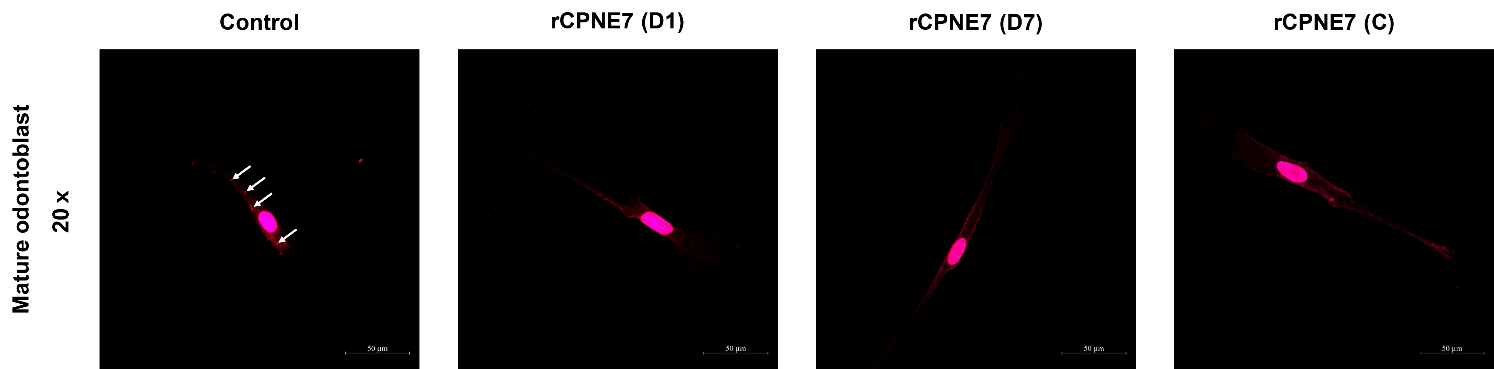
**

**Supplementary Figure 3.** CPNE7 removed lipofuscin in mature odontoblasts. hDPC were differentiated for 21 days with rCpne7 treatment for the following(indicated) period of time. At mature stage (D1; 20 – 21 days), secretory to mature stage (D7; 14 – 21 days), whole differentiation stage (C; continuously, 0 – 21 days) of odontoblast. Lipofuscin (red dots; white arrows) was observed by immunofluorescence. DAPI (blue) was counterstained to indicate the nucleus. Scale bars: 50 μm.
